# Supplementary material for: Nanobody-based VSR7 tracing shows clathrin-dependent TGN to Golgi recycling
Source: Nat Commun. 2023 Oct 30;14:6926. doi: 10.1038/s41467-023-42331-1 (PMC10616157; doi:10.1038/s41467-023-42331-1)
Supplement: Supplementary file 1 — Supplementary Information [file 41467_2023_42331_MOESM1_ESM.pdf]

Nanobody-based VSR7 tracing shows clathrin-dependent TGN to Golgi recycling.

Xiaoyu Shao[1, 2, 3], Hao Xu[2, 3] and Peter Pimpl[2, 3*]

[1]Harbin Institute of Technology, Harbin 150001, China
[2]Key Laboratory of Molecular Design for Plant Cell Factory of Guangdong Higher Education Institutes, Southern University of Science and Technology (SUSTech), Shenzhen, Guangdong 518055, China
[3]Institute of Plant and Food Science, School of Life Sciences, Department of Biology, Southern University of Science and Technology (SUSTech), Shenzhen, Guangdong 518055, China
*For correspondence: pimpl@sustech.edu.cn.

Supplementary information

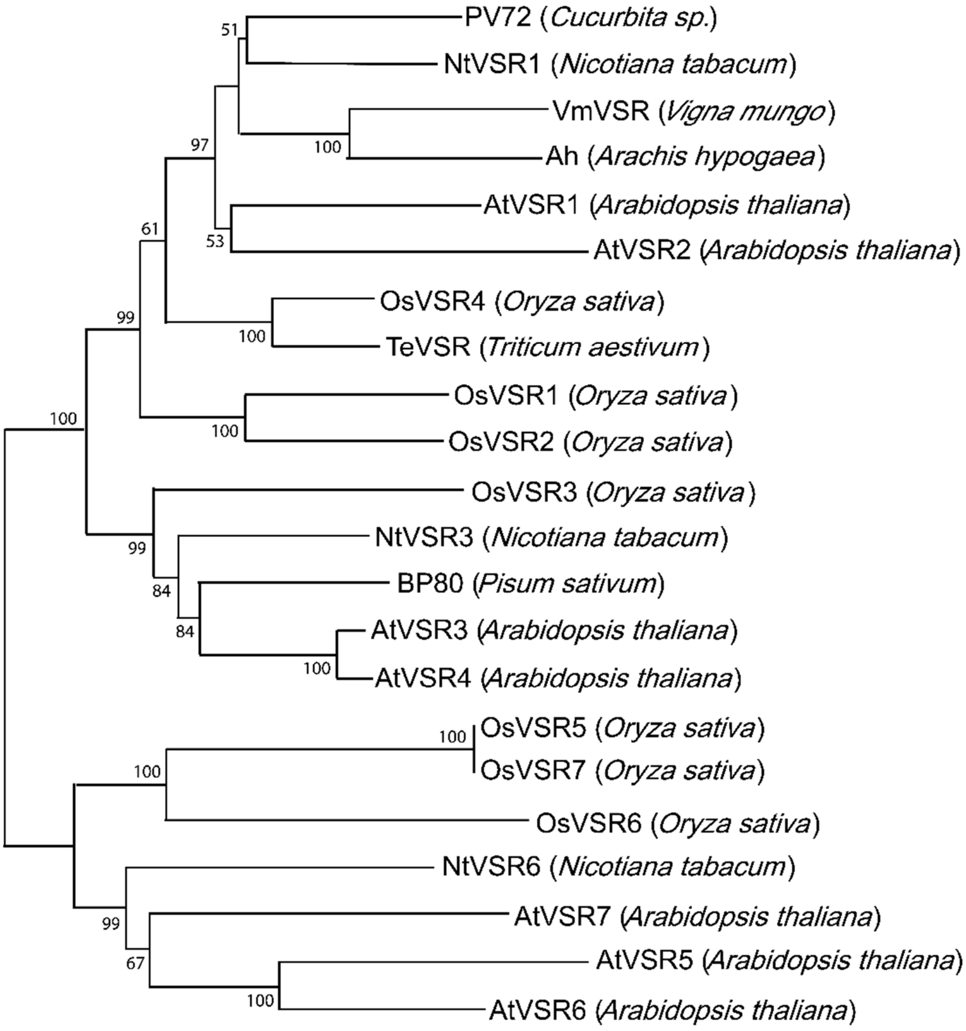

Supplementary Figure 1 Neighbor-joining tree of proteins encoded by VSR genes. The tree summarizes the evolutionary relationship of VSR proteins from different plant species, which can be divided into two major subclades that are represented by the class I and II VSRs VSR1, VSR2, VSR3, and VSR4, and the class III VSRs VSR5, VSR6, and VSR7, with the VSR7 as being the most distant member of the family. Three VSRs have been described in *Nicotiana tabacum*: the class I NtVSR1, the class II NtVSR3, and the class III NtVSR6. Amino acid sequences of the respective proteins are given in the Source Data file.

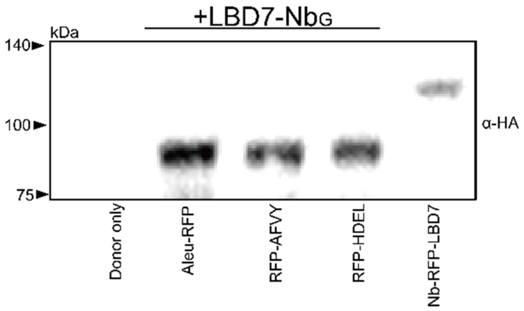

Supplementary Figure 2 Expression control of the sensing unit of the VSR7 sensor. SDS-PAGE/WB analysis showing the expression of the HA-tagged sensing unit LBD7-Nb_G in coexpression with the ER anchor GFP-CNX and the respective RFP-fusion proteins used for the FLIM analysis shown in Fig. 1e, using anti-HA antibodies. The experiment was repeated twice with similar results, and the uncropped images are shown in Supplementary Figure 9.

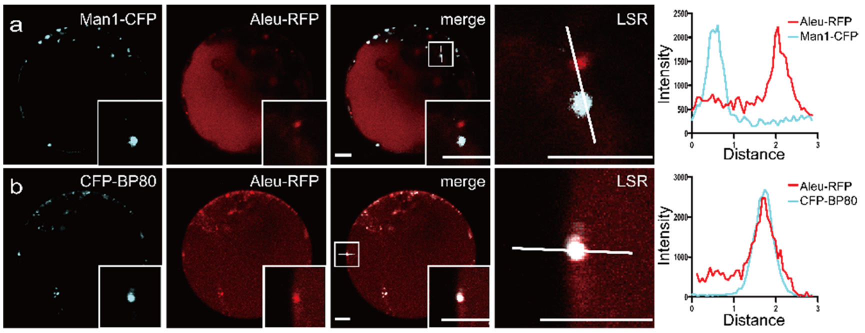

Supplementary Figure 3 Punctate Aleu-RFP signals represent MVBs/LEs. **a** Coexpression of Aleu-RFP (red) with the *cis*-Golgi marker Man1-CFP (blue) does not result in colocalization of signals, as was previously shown[1]. **b** Coexpression of Aleu-RFP (red) with the MVB/LE marker CFP-BP80 (blue) shows colocalization of the punctate signals, as was previously shown[1]. Insets are magnifications of the region indicated by a white square in the merged image. LSR, line scan region of the line intensity analysis. Scale bars = 5 µm. The experiments were repeated thrice with similar results, and representative images are shown. Source data for **a**, **b** are provided as a Source Data file.

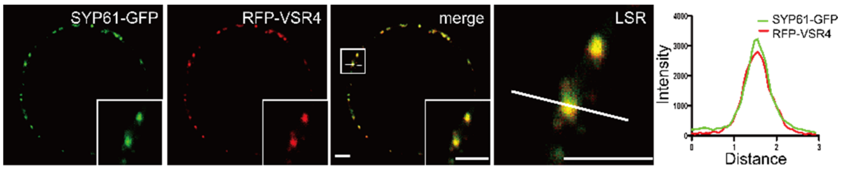

Supplementary Figure 4 The fluorescent full-length VSR4 localizes at the TGN/EE. Coexpression of the fluorescent full-length VSR4, RFP-VSR4 (red), with the TGN/EE marker SYP61-GFP (green) shows overlapping signals and thus TGN/EE localization of the receptor, as was previously shown[2]. Insets are magnifications of the region indicated by a white square in the merged image. LSR, line scan region of the line intensity analysis. Scale bars = 5 µm. The experiment was repeated thrice with similar results, and representative images are shown. Source data are provided as a Source Data file.

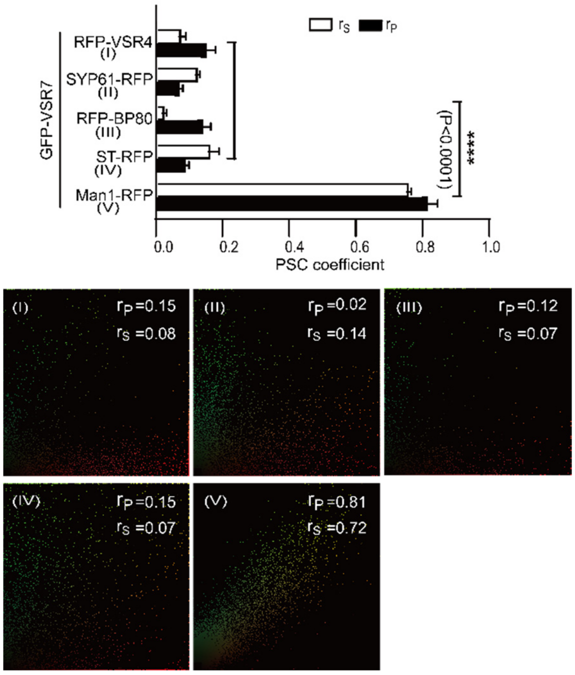

Supplementary Figure 5 Quantification of the VSR7 localization analysis. Pearson's (r_P) and Spearman's (r_S) correlation (PSC) coefficients for the protein combinations indicated by the Roman numerals were calculated for n=10 cells; averages are shown in the chart, with error bars indicating SD. A representative scatter plot of the analysis from a single cell is shown below the chart for each of the above combinations. Source data are provided as a Source Data file. Statistical analysis of the PSC coefficients of GFP-VSR7 and (I) RFP-VSR4 (shown in Fig. 3a), (II) SYP61-RFP (shown in Fig. 3b), (III) RFP-BP80 (shown in Fig. 3c), (IV) ST-RFP (shown in Fig. 3d), or (V) Man1-RFP (shown in Fig. 3e) demonstrates that GFP-VSR7 colocalizes with the *cis*-Golgi marker Man1-RFP, but not with the TGN/EE-localizing VSR4, the TGN/EE marker SYP61-RFP, the MVB/LE marker RFP-BP80, or the *trans*-Golgi marker ST-RFP. Significance was calculated using two-way ANOVA, followed by Student's t-test compared with the other group. The experiment was repeated twice with similar results.

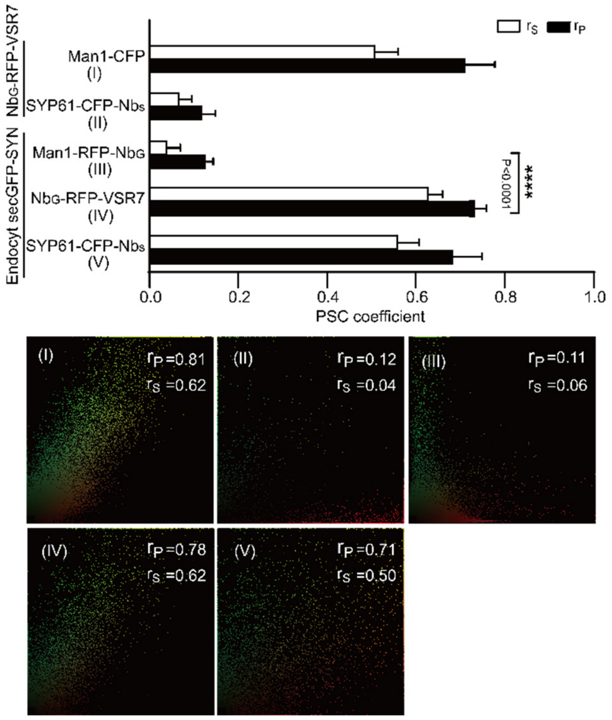

Supplementary Figure 6 Quantification of the colocalization of endocytosed secGFP-SYN with the Golgi-localizing proteins Nb$_G$-RFP-VSR7 and ManI-RFP-Nb$_G$. Pearson's (r_P) and Spearman's (r_S) correlation (PSC) coefficients for the protein combinations indicated by the Roman numerals were calculated for n=10 cells; averages are shown in the chart, with error bars indicating SD. A representative scatter plot of the analysis from a single cell is shown below the chart for each of the above combinations. Source data are provided as a Source Data file. Analysis of the PSC coefficients of the Nb$_G$-RFP-VSR7 and (I) Man1-CFP (shown in Fig. 4b) demonstrate the colocalization of the Nb$_G$-tagged receptor with the *cis*-Golgi marker, or (II) SYP61-CFP-Nb$_S$ (shown in Fig. 4e) demonstrates that Nb$_G$-RFP-VSR7 does not colocalize with the TGN/EE marker. Statistical analysis of the PSC coefficients of the endocytosed dual-epitope linker secGFP-SYN and (III) the Man1-RFP-Nb$_G$ (shown in Fig. 4d), and (IV) Nb$_G$-RFP-VSR7 (shown in Fig.4f), demonstrate that the endocytosed secGFP-SYN does not colocalize with the Nb$_G$-tagged *cis*-Golgi marker Man1-RFP-Nb$_G$ but colocalizes with the *cis*-Golgi-localizing Nb$_G$-tagged VSR7, Nb$_G$-RFP-VSR7, and (V) SYP61-CFP-Nb$_S$ (shown in Fig. 4f), demonstrates the colocalization with the Nb$_S$-tagged TGN/EE marker SYP61-CFP-Nb$_S$. Significance was calculated using two-way ANOVA, followed by Student's t-test. The experiment was repeated twice with similar results.

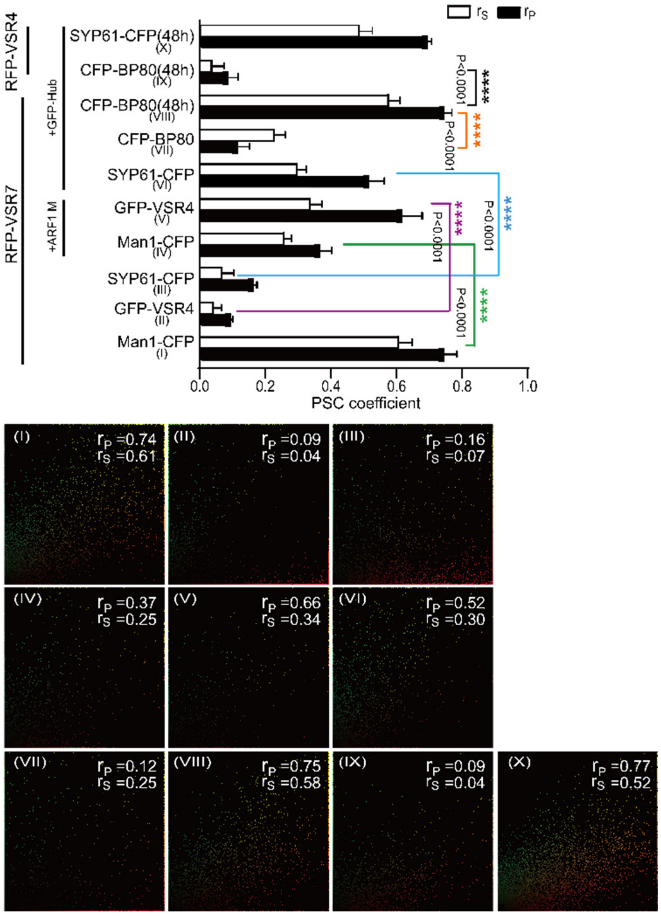

Supplementary Figure 7 Quantification of the VSR7 recycling analysis reveals ARF1 and clathrin dependency. Pearson's (r_P) and Spearman's (r_S) correlation (PSC) coefficients for the protein combinations indicated by the Roman numerals were calculated for n=10 cells; averages are shown in the chart, with error bars indicating SD. A representative scatter plot of the analysis from a single cell is shown below the chart for each of the above combinations. Source data are provided as a Source Data file. Statistical analysis of the comparison of PSC coefficients of RFP-VSR7 and (I) Man1-CFP or (II) GFP-VSR4 (shown in Fig.5b), or (III) SYP61-CFP (shown in Supplementary Fig. 8) of imaged control cells with the PSC coefficients of RFP-VSR7 and (IV) Man1-CFP (shown in Fig. 5a) or (V) GFP-VSR4 (shown in Fig. 5c) in the presence of the ARF1 mutant, or (VI) SYP-61-CFP (shown in Fig. 5d), or (VII) CFP-BP80 (shown in Fig. 5e) in the presence of the GFP-Hub, or (VIII) CFP-BP80 after 48 h (shown in Fig. 5f). Statistical analysis of PSC coefficients of RFP-VSR4 and (IX) CFP-BP80 after 48 h (shown in Fig. 5g) or SYP61-CFP after 48 h (shown in Fig. 5h) in the presence of the GFP-Hub. In control cells, the RFP-VSR7 colocalizes with the *cis*-Golgi marker Man1-CFP but not with the TGN/EE-localizing GFP-VSR4 or the TGN/EE marker SYP61-CFP. Expression of the ARF1 mutant significantly reduces the colocalization between the RFP-VSR7 and the *cis*-Golgi marker Man1-CFP (green) and significantly increases the colocalization with the GFP-VSR4 (purple). The coexpression of the GFP-Hub significantly increases the colocalization of the RFP-VSR7 with the TGN/EE marker SYP61-CFP (blue). Prolonged expression of the GFP-Hub for 48 hours causes the RFP-VSR7 to colocalize with the MVB/LE marker CFP-BP80 (orange), while RFP-VSR4 colocalizes with the TGN/EE marker SYP61-CFP, but not with the MVB/LE marker CFP-BP80. Significance was calculated using two-way ANOVA, followed by Student's t-test. The experiment was repeated twice with similar results.

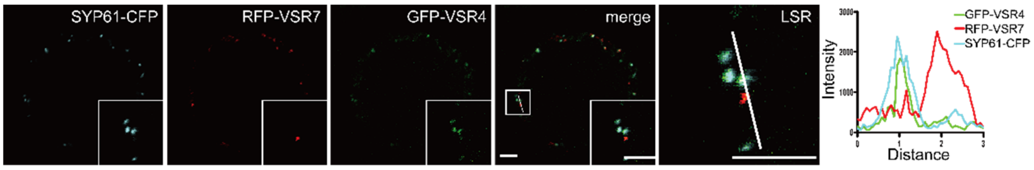

Supplementary Figure 8 RFP-VSR7 does not colocalize with GFP-VSR4 at the TGN/EE. Coexpression of the RFP-VSR7 with GFP-VSR4, and SYP61-CFP results in no colocalization between the signals from RFP-VSR7 and SYP61-CFP and GFP-VSR4, respectively. Insets are magnifications of the region indicated by a white square in the merged image. LSR, line scan region of the line intensity analysis. Scale bars = 5 µm. The experiment was repeated thrice with similar results, and representative images are shown. Source data are provided as a Source Data file.

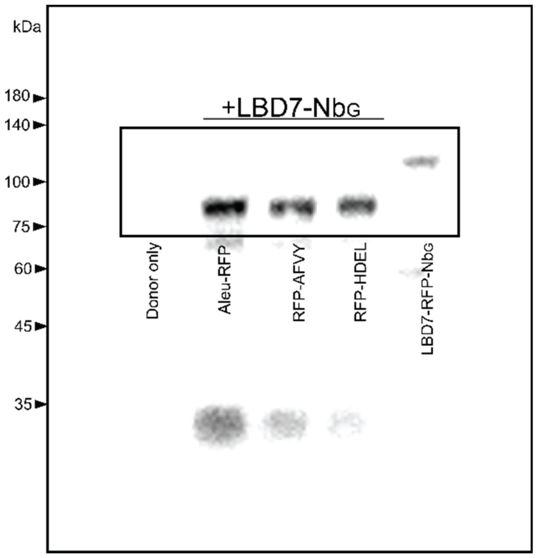

Supplementary Figure 9 Uncropped image of the SDS-PAGE/WB analysis from Supplementary Fig. 2

Supplementary Table 1 Genetic constructs generated in this study

	Primers	Sequence (5'-3')	Template	Recipient Vector
LBD7-RFP-Nb$_G$ (pAZ05)	LBD7-*Nhe*I-S	CAGTAGGCTAGCATGGGTTTAGTCAACGGGAGA	First-strand cDNA from 7-day-old *Arabidopsis* seedlings	pSF065[2] cut *Nhe*I/*Not*I
	LBD7- *Not*I -AS	ACAAGGGCGGCCGCTGCCGTTTTGGATCCATA		
LBD7-Nb$_G$ (pNW03)	LBD7 (*Nhe*I/*Bam*HI), subcloned from pAZ05			pS06 cut *Nhe*I/*Not*I
GFP-VSR7 (pXY263)	VSR7-*Not*I-S	ACAAGGGCGGCCGCAGGTTTGTGGTGGAGAAAGAAAGC	First-strand cDNA from 7-day-old *Arabidopsis* seedlings	pXY 255 cut *Not*I/*BamH*I
	VSR7-*BamH*I-AS	CTGCTTCGGATCCTTAGAGTGAAAAGGCTCGGCTTCTG		
Nb$_G$-RFP-VSR7 (pXH 03)	VSR7 (*Nde*I/*BamH*I), subcloned from pXY263			pSF075 cut *Nde*I/*Bam*HI
RFP-HDEL (pXY229)	RFP-*Cla*I-S	AGTCTAATCGATGAGGCTTTGTAAATTCACAGCTC	pSF084[2]	cut *Cla*I/*Bam*HI
	RFP-HDEL-*Bam*HI -AS	AGTCTAGGATCCCTAAAGCTCATCATGTGCTCCAGTACTGTGGCG		
RFP-AFVY (pXY245)	RFP-*Nhe*I-S	CAGTACGCTAGCATGGCCTCCTCCGAGGACGTC	pSF084[2]	pFK 089[3] cut *Nhe*I/*Bam*HI
	RFP-AFVY-*Bam*HI -AS	TGCTTCGGATCCTTAGTACACAAAGGCTGCTCCAGTACTGTGGCGGC		
Man1-RFP-Nb$_G$ (pXY 264)	Nb$_G$ (*Cla*I/*Bam*HI), subcloned from pXY263			pSF150[2] cut *Cla*I/*Bam*HI

Supplementary Table 2 Marker and reporter used in this study

Name	Function
GFP-CNX[1] (pFF04)	ER marker
Man1- GFP/CFP/BFP2[4]	*cis*-Golgi marker
ST-RFP/GFP[1]	*trans*-Golgi marker
SYP61-GFP/RFP/CFP[1]	TGN/EE marker
RFP/CFP-BP80[2]	MBV/LE marker
Aleu-RFP[1]	MVB/LE and vacuolar marker, VSR ligand
SYP61-RFP-Nb[2] (pSF80)	Nanobody-tagged fluorescent TGN/EE marker/anchor
Nb$_G$-RFP-VSR4 (pSF076)	Nanobody-tagged fluorescent VSR4
GFP-SYN[2] (pSF74)	Dual-epitope linker protein
RFP-VSR4[2] (pSF47)	Fluorescent VSR4
GFP-Hub[5]	Fluorescent dominant-negative clathrin mutant to inhibit clathrin-mediated transport in eukaryotic cells
ARF1M[6]	GTP-locked dominant-negative ARF1 mutant

References

1 Künzl, F., Früholz, S., Fässler, F., Li, B. & Pimpl, P. Receptor-mediated sorting of soluble vacuolar proteins ends at the trans-Golgi network/early endosome. *Nat Plants* **2**, 16017, doi:10.1038/nplants.2016.17 (2016).

2 Früholz, S., Fässler, F., Kolukisaoglu, U. & Pimpl, P. Nanobody-triggered lockdown of VSRs reveals ligand reloading in the Golgi. *Nature communications* **9**, 643, doi:10.1038/s41467-018-02909-6 (2018).

3 Robinson, D. G. & Neuhaus, J. M. Receptor-mediated sorting of soluble vacuolar proteins: myths, facts, and a new model. *J Exp Bot* **67**, 4435-4449, doi:10.1093/jxb/erw222 (2016).

4 Nebenfuhr, A., Gallagher, L. A., Dunahay, T. G., Frohlick, J. A., Mazurkiewicz, A. M., Meehl, J. B. & Staehelin, L. A. Stop-and-go movements of plant Golgi stacks are mediated by the acto-myosin system. *Plant Physiol.* **121**, 1127-1142 (1999).

5 Scheuring. D., V. C., Krüger. F., Künzl. F.,Sturm. S.,Bubeck. J., Hillmer. S., Frigerio. L., Robinson. D. G., Pimpl. P., and Schumacher. K. Multivesicular bodies mature from the trans-Golgi network/early endosome in Arabidopsis. *The Plant Cell* **23**, 3463-3481 (2011).

6 Peter Pimpl, S. L. H., J. Philip Taylor, Luis L. Pinto-daSilva, and Jürgen Denecke The GTPase ARF1p controls the sequence-specific vacuolar sorting route to the lytic vacuole.pdf>. *Plant Cell* **15**, doi:10.1105/tpc.010140 (2003).
