# Supplementary material for: Nanobody-based VSR7 tracing shows clathrin-dependent TGN to Golgi recycling
Source: Nat Commun. 2023 Oct 30;14:6926. doi: 10.1038/s41467-023-42331-1 (PMC10616157; doi:10.1038/s41467-023-42331-1)
Supplement: Supplementary file 3 — Reporting Summary [file 41467_2023_42331_MOESM3_ESM.pdf]

nature portfolio

Corresponding author(s): Peter Pimpl

Last updated by author(s): Sep 19, 2023

Reporting Summary

Nature Portfolio wishes to improve the reproducibility of the work that we publish. This form provides structure for consistency and transparency in reporting. For further information on Nature Portfolio policies, see our Editorial Policies and the Editorial Policy Checklist.

Statistics

For all statistical analyses, confirm that the following items are present in the figure legend, table legend, main text, or Methods section.

n/a	Confirmed	
☐	☒	The exact sample size (*n*) for each experimental group/condition, given as a discrete number and unit of measurement
☐	☒	A statement on whether measurements were taken from distinct samples or whether the same sample was measured repeatedly
☐	☒	The statistical test(s) used AND whether they are one- or two-sided<br>*Only common tests should be described solely by name; describe more complex techniques in the Methods section.*
☒	☐	A description of all covariates tested
☒	☐	A description of any assumptions or corrections, such as tests of normality and adjustment for multiple comparisons
☐	☒	A full description of the statistical parameters including central tendency (e.g. means) or other basic estimates (e.g. regression coefficient) AND variation (e.g. standard deviation) or associated estimates of uncertainty (e.g. confidence intervals)
☐	☒	For null hypothesis testing, the test statistic (e.g. *F*, *t*, *r*) with confidence intervals, effect sizes, degrees of freedom and *P* value noted<br>*Give P values as exact values whenever suitable.*
☒	☐	For Bayesian analysis, information on the choice of priors and Markov chain Monte Carlo settings
☒	☐	For hierarchical and complex designs, identification of the appropriate level for tests and full reporting of outcomes
☐	☒	Estimates of effect sizes (e.g. Cohen's *d*, Pearson's *r*), indicating how they were calculated

Our web collection on statistics for biologists contains articles on many of the points above.

Software and code

Policy information about availability of computer code

Data collection	SymphoTime64 v2.0, PicoQuant GmbH, Berlin, Germany
Data analysis	SymphoTime64 v2.0, PicoQuant GmbH, Berlin, Germany.<br>ImageJ v.1.51 (https://imagej.nih.gov/ij/index.html).<br>GraphPad Prism 8.0 (https://www.graphpad.com/scientific-software/prism).<br>ClustalW (https://www.ebi.ac.uk/clustalw).<br>Molecular Evolutionary Genetics Analysis (MEGA) version 11 (https://www.megasoftware.net/).<br>G*Power Version 3.1.9.2 (https://www.psychologie.hhu.de/arbeitsgruppen/allgemeine-psychologie-und-arbeitspsychologie/gpower).

For manuscripts utilizing custom algorithms or software that are central to the research but not yet described in published literature, software must be made available to editors and reviewers. We strongly encourage code deposition in a community repository (e.g. GitHub). See the Nature Portfolio guidelines for submitting code & software for further information.

Data

Policy information about availability of data

All manuscripts must include a data availability statement. This statement should provide the following information, where applicable:

- Accession codes, unique identifiers, or web links for publicly available datasets
- A description of any restrictions on data availability
- For clinical datasets or third party data, please ensure that the statement adheres to our policy

All data supporting the findings of this study are available within the paper and its Supplementary Information.

Research involving human participants, their data, or biological material

Policy information about studies with human participants or human data. See also policy information about sex, gender (identity/presentation), and sexual orientation and race, ethnicity and racism.

Reporting on sex and gender	-
Reporting on race, ethnicity, or other socially relevant groupings	-
Population characteristics	-
Recruitment	-
Ethics oversight	-

Note that full information on the approval of the study protocol must also be provided in the manuscript.

Field-specific reporting

Please select the one below that is the best fit for your research. If you are not sure, read the appropriate sections before making your selection.

☒ Life sciences ☐ Behavioural & social sciences ☐ Ecological, evolutionary & environmental sciences

For a reference copy of the document with all sections, see nature.com/documents/nr-reporting-summary-flat.pdf

Life sciences study design

All studies must disclose on these points even when the disclosure is negative.

Sample size	The sample size was estimated based on previously achieved effect sizes in our lab: Effect size f (ANOVA) for the data shown in Figure 1e was 1.24; with our desired error values (=0.001, (1-β)=0.95) and 5 different groups of samples, this computes to a minimum of 35 total samples or 7 samples per group. Effect size f (ANOVA) for the data shown in Figure 2e was 0.96; with our desired error values (=0.001, (1-β)=0.95) and first 4 different groups of an absence of the drug samples, the other 4 different groups of samples in the presence of the Conc A drug, this computes to a minimum of 17 total samples or 5 samples per group. For Supplementary Fig 5, Supplementary Fig 6, and Supplementary Fig 7, for each of the images we used for analysis, the threshold was set to 10. Effect size f (ANOVA) for the data shown in Supplementary Fig 5 was 1.125, with our desired error values (=0.001, (1-β)=0.95) and 5 different groups of samples, this computes to a minimum of 25 otal samples or 7 samples per group. Effect size f (ANOVA) for the data shown in Supplementary Fig 6, and Supplementary Fig 7 was 7.54, in total, we chose 5 different groups in S6, and 10 different groups in S7, with our desired error values (=0.001, (1-β)=0.95) of 2 different groups for comparison, this computes to a minimum of 150 total samples or 10 samples per group. The calculation was performed using G*Power Version 3.1.9.2. We increased this number to 10 samples per group to accommodate for possible slightly weaker effect sizes or data point distributions, which would necessitate alternative non-parametric tests.
Data exclusions	Protoplast that were not completely turgescent or exhibited any signs of damage were excluded from image acquisition. For FRET-FLIM analysis ROIs were chosen in a way, that all fluorescent signals, which did not obviously originate from Chlorophyll autofluorescence, were used to calculate average lifetimes.<br>The Peason's correlation coefficient and Spearman's correlation for the colocalization/no colocalization statistical analysis, threshold were set to be 10 as a single group.
Replication	All attempts at replication were successful.
Randomization	All protoplasts for an experiments were taken from a single pool, allocation into different transformations samples was performed by pipetting ~3*10^6 at once. Thus individual cells were completely randomly distributed to the transformation samples.
Blinding	Blinding was not performed. Due to characteristic distribution of fluorescently tagged proteins in the cells, the investigators are able to deduct which sample they are observing.

Reporting for specific materials, systems and methods

We require information from authors about some types of materials, experimental systems and methods used in many studies. Here, indicate whether each material, system or method listed is relevant to your study. If you are not sure if a list item applies to your research, read the appropriate section before selecting a response.

Materials & experimental systems

n/a	Involved in the study	
	☒	Antibodies
☒		Eukaryotic cell lines
☒		Palaeontology and archaeology
	☒	Animals and other organisms
☒		Clinical data
☒		Dual use research of concern
	☒	Plants

Methods

n/a	Involved in the study	
☒		ChIP-seq
☒		Flow cytometry
☒		MRI-based neuroimaging

Antibodies

Antibodies used	rat monoclonal anti-HA–Peroxidase (Roche 12013819001, 1:5,000)
Validation	https://www.sigmaaldrich.com/deepweb/assets/sigmaaldrich/product/documents/348/595/12013819001.pdf

Animals and other research organisms

Policy information about studies involving animals; ARRIVE guidelines recommended for reporting animal research, and Sex and Gender in Research

Laboratory animals	-
Wild animals	-
Reporting on sex	-
Field-collected samples	-
Ethics oversight	-

Note that full information on the approval of the study protocol must also be provided in the manuscript.

Dual use research of concern

Policy information about dual use research of concern

Hazards

Could the accidental, deliberate or reckless misuse of agents or technologies generated in the work, or the application of information presented in the manuscript, pose a threat to:

No	Yes	
☒	☐	Public health
☒	☐	National security
☒	☐	Crops and/or livestock
☒	☐	Ecosystems
☒	☐	Any other significant area

Experiments of concern

Does the work involve any of these experiments of concern:

No	Yes	
☒	☐	Demonstrate how to render a vaccine ineffective
☒	☐	Confer resistance to therapeutically useful antibiotics or antiviral agents
☒	☐	Enhance the virulence of a pathogen or render a nonpathogen virulent
☒	☐	Increase transmissibility of a pathogen
☒	☐	Alter the host range of a pathogen
☒	☐	Enable evasion of diagnostic/detection modalities
☒	☐	Enable the weaponization of a biological agent or toxin
☒	☐	Any other potentially harmful combination of experiments and agents
